# Antioxidant, Transcriptome and the Metabolome Response to Dietary Astaxanthin in *Exopalaemon carinicauda*

**DOI:** 10.3389/fphys.2022.859305

**Published:** 2022-03-30

**Authors:** Wenyang Li, Jiajia Wang, Jitao Li, Ping Liu, Jian Li, Fazhen Zhao

**Affiliations:** ^1^Wuxi Fisheries College, Nanjing Agricultural University, Nanjing, China; ^2^Laboratory for Marine Fisheries Science and Food Production Processes, Pilot National Laboratory for Marine Science and Technology (Qingdao), Yellow Sea Fisheries Research Institute, Chinese Academy of Fishery Sciences, Qingdao, China

**Keywords:** astaxanthin, antioxidant, *Exopalaemon carinicauda*, transcriptome, metabolome

## Abstract

Astaxanthin (Axn), a feed additive, is becoming increasingly important for modulating the metabolism, growth, development, and reproduction of aquatic organisms in aquaculture. In this study, *Exopalaemon carinicauda* (*E. carinicauda*) is an economically important fishery species in China that has been found to exhibit increased body weight following Axn feeding as compared to a standard diet. The antioxidant, transcriptomic, and metabolomic analyses of the response of *E. carinicauda* after Axn feeding were investigated. Axn could reduce the content of malondialdehyde and increase the activities of various antioxidant enzymes, which also proved that axn can improve the antioxidant capacity Transcriptomic analysis suggested that synthesis and secretion of immune proteins, cytoskeleton structure, and apoptosis signaling were altered after Axn feeding. The metabolic response to axn mainly includes the up regulation of different amino acids and the change of unsaturated fatty acids. Combined transcriptomic and metabolomic data indicated that amino acid metabolic pathways were upregulated in the muscles after Axn feeding. For good measure, energy metabolism pathways were upregulated in the muscles to improve ATP and unsaturated fatty acid production. This study provides key information to increase our understanding of the effects of Axn in shrimp.

## Introduction

The marine carotenoid astaxanthin (Axn) is naturally found in a wide variety of aquatic organisms, such as microalgae, crustaceans (crabs, lobsters, and shrimp), and fish (salmon and trout) (Mattei et al., 2011; Albrektsen et al., 2018), but cannot be synthesized by most aquatic animals from scratch. Its *de novo* synthesis is limited to several bacteria, protists, fungi, algae, and plants, and most of the natural Axn used as an aquatic feed additive is extracted from *Haematococcus pluvialis* (Fang et al., 2019). Axn not only exhibits a potent antioxidant function as a powerful scavenger of oxygen-free radicals, but can also enhance resistance to different types of environmental stresses, including salinity, oxygen depletion, and high temperature (Yu et al., 2020). More importantly, Axn protects against oxidative stress by scavenging free radicals and neutralizing singlet oxygen and cytokine production and plays a key role in the anti-inflammatory response by regulating the NF-κB and apoptosis pathways (Fakhri et al., 2018; Xie et al., 2018; Li et al., 2019). Therefore, Axn, a significant antioxidant, can be used to improve the growth performance and enhance the stress tolerance of marine species.

The ridgetail white prawn (*E. carinicauda*) is an important commercial polyculture species in the coastal area of Jiangsu, Zhejiang, Hebei and Shandong Provinces of China. At present, the yield of ridgetail white prawn accounts for one third of the total amount of mixed culture ponds along the eastern coast of China (Xu et al., 2010; Zhang et al., 2018). In addition to its multiple advantages such as short reproductive cycle and rapid growth, this shrimp is also of great significance in environmental adaptation and stress resistance (Ge et al., 2017).

The rapid development of biotechnology can promote the research on the reaction mechanism of marine organisms feeding astaxanthin, so as to deepen the understanding of the molecular mechanism of feeding astaxanthin. Transcriptomics, comprising high-throughput data generation and functional analyses, can help determine the major influence of various factors in aquatic organisms (Wen et al., 2019), and identify the important genes and signaling pathways involved in the effects of Axn feeding. However, its disadvantage is that the phenotype is indirectly influenced by changes in mRNA expression (Sun et al., 2019), while metabolomics provides intuitive data of tissue phenotypes under particular environmental conditions at a specified time (Huo et al., 2019). Thus, metabolomics must be performed along with transcriptomics (May, 2017). The analysis of a combination of gene expression and metabolomics data can provide system-level snapshots of the metabolism of an organism during Axn feeding (Ning et al., 2019). However, there has been no study on the use of multiomics in investigating the mechanisms of the muscle responses of *E. carinicauda* to Axn feeding.

It has been reported that dietary supplementation with Axn can improve the immune capacity and low salinity tolerance of *Litopenaeus vannamei* (*L. vannamei*) (Xie et al., 2018); however, there is no research evaluating the effect of dietary Axn supplementation on the muscle of *E. carinicauda*. In this study, transcriptomic and metabolomic analyses were used to investigate the major pathways responsible for the molecular responses to Axn feeding, with the aim of revealing the mechanism by which the muscle of *E. carinicauda* responds to Axn feeding. Malondialdehyde (MDA) and antioxidant enzymes were used to verify the main molecular response pathway in the muscle of *E. carinicauda*. It is clear that combining metabolomics, transcriptomics, and functional analyses may represent a new approach to understanding the response mechanisms of *E. carinicauda* to Axn feeding.

## Materials and Methods

### Experimental Diets

Commercial shrimp feed with an approximate diameter of 1.0 mm for *L. vannamei* without Axn supplementation was used as the basal diet. Axn (Bioalgo, Shandong, China) was supplemented into the basal diet at a dose of 0.1 g kg^–1^, which was determined as the optimal dose in our pre-experiment. The feed formulation was shown in Table 1.

**TABLE 1:** The composition of the base feed (g kg^–1^).

Composition	Group and content	
	Control group	Axn group
Fish meal	190	190
Soybean meal	281	281
Soybean oil	15	15
Fish oil	5	5
Complex vitamin	12	12
Complex mineral	17	17
Axn	\	0.1

*Composition of multivitamin (kg^–1^): VA, 300,000 IU; VB2, 480 mg; VB6, 360 mg; B12, 1.2 mg; VB1, 20.0 mg; Vitamin k, 20 mg; Folic acid, 170 mg; Biotin, 10 mg; VE, 3,000 IU; Inositol, 8,000 mg; Calcium pantothenate, 800 mg; Niacin, 200 mg; Choline chloride, 8,000 mg; VD, 40,000 IU. Composition of complex minerals (kg^–1^ feed): ZnSO_4_⋅7H_2_O, 0.817 g; CaCO_3_, 3.28 g; NaH_2_PO_4_, 2.96 g; KH_2_PO_4_, 6.752 g; CaCl_2_, 1.3328 g; MgSO_4_⋅7H_2_O, 1.6 g; KCl, 0.448 g; AlCl_3_⋅6H_2_O, 0.0192 g; MnSO_4_⋅(4/6) H_2_O, 0.229 g; CuCl_2_, 0.52 g; FeSO_4_⋅7H_2_O, 1.8 g; CoCl_2_, 0.0282 g; KI, 0.031 g.*

### Experimental Shrimps and Sampling

Juvenile *E. carinicauda* was obtained from a shrimp hatchery in Rizhao, China. During a week of acclimation, the animals were maintained in an indoor cement pool and fed with first hatched brine shrimp (Artemia salina) three times a day, and approximately half of the seawater in the pool was renewed once a day. A total of 180 healthy shrimps (0.06 ± 0.01 g) were randomly assigned to six tanks filled with 30 L sand-filtered seawater. Each treatment was randomly assigned to three replicated tanks with 30 shrimp each. Shrimp were fed three times a day at 07:00, 12:00, and 18:00 with a daily ration of approximately 5% body mass for 8 weeks. When approximately 50% of seawater in the tanks was renewed, the feces, uneaten feed, and exuviae were removed. Natural illumination was used during the feeding trial, and water quality was maintained at a salinity of 30 ± 1, temperature of 23 ± 0.5°C, pH of 8.2 ± 0.1, and dissolved oxygen of 7.4 ± 0.3 mg L^–1^.

All the tanks were used to pool samples that split into two replicate groups of three tanks. The muscles of the two groups were taken after 8 weeks. At each of the two groups, three shrimps were taken from each tank (total = 18 shrimps) for transcriptome analysis and six shrimps were taken from each tank (total = 36 shrimps) for metabonomics analysis. Whole muscles samples were immediately flash frozen in liquid nitrogen, and stored at −80°C until further analysis.

### Biochemical Assay and Growth Performances

Three muscles per treatment were weighed and homogenized in pre-chilled 0.86% saline solution (1:9, w/v) and centrifuged, and the supernatant was collected to measure total protein content, MDA content, superoxide dismutase (SOD), catalase (CAT), glutathione (GSH), and total antioxidant capacity (T-AOC). All biochemical parameters were determined using commercial assay kits (Nanjing Jiancheng Bioengineering Institute, Nanjing, China) according to the manufacturer’s instructions. Differences in biochemical parameters were considered statistically significant at *P* < 0.05 using a *t*-test. Data are expressed as means ± SD (*n* = 3).

Final body weight (FBW), final body weight (g); WG, weight gain (%) = [(final body weight - initial body weight)/initial body weight] × 100.SGR, specific growth rate (%) = [(loge final body weight - loge initial body weight)/days] × 100.

### Transcriptomic Analysis

Total RNA was isolated from muscle tissue using TRIzol reagent (Takara, Japan). The isolated RNA was quantified and qualified using a NanoPhotometer^®^ spectrophotometer (IMPLEN, Westlake Village, CA, United States) and Qubit^®^ RNA Assay Kit in Qubit^®^2.0 Fluorometer (Life Technologies, Carlsbad, CA, United States), respectively. RNA integrity was detected using 1% agarose gels and an RNA Nano 6000 Assay Kit of the Agilent Bioanalyzer 2100 system (Agilent Technologies, Santa Clara, CA, United States). Sequencing libraries were constructed using the NEBNext^®^ Ultra™ RNA Library Prep Kit for Illumina^®^ (New England Biolabs, Ipswich, MA, United States). All experiments were performed in accordance with the manufacturer’s protocol.

### Gene Validation From the Transcriptome Data

Nine differentially regulated mRNAs from the Illumina sequencing results were validated using quantitative real-time reverse transcription polymerase chain reaction (qRT-PCR). Primers were designed using Primer Premier 5 for qRT-PCR. Table 2 lists the primers used. mRNA expression was analyzed by qRT-PCR, and *18S rRNA* was used as an internal control. The SYBR Premix Ex Taq on the ABI PRISM 7500 Sequence Detection System (Applied Biosystems, Thermo Fisher Scientific, Waltham, MA, United States) was used to perform qRT-PCR. The PCR program was as follows: 95°C for 10 min, followed by 45 cycles at 95°C for 15 s and 60°C for 30 s. The fold change in the expression levels of the target genes was calculated using the relative quantitative method (2^–ΔΔCt^).

**TABLE 2:** Genes and primer sequences used in gene expression validation experiments.

Gene	Forward primer (5′-3′)	Reverse primer (5′-3′)
V-ATP	ATGCGAAAACGACAGATC CAGGTAC	GGCAGCAGAAGATCCCACTATTCC
eIF2α	CGAAGTGGACGATGTGG TGATGG	CGGGACAGTTCTGACAGCAGTATC
HSP90	GCACCTGCTCACGAGA TTCACC	ATTTCCTGCGTTACCACACCTCTTC
HSP70	TCACAGACACAGAACGCC TTATTGG	GGACAACGCCATCATCAAACTTTCG
c-jun	GCAGCGGCAGTATCGT GGTAAC	CTGTAGATGCGGATGATGGTGGTG
Cathepsin	CGACGCCTGTGCCT CCTTTATG	TCTTCACGAGCCAGTAGTCCATACC
Bcl-XL	GCCACGCTAACGAAGGA CATATACC	CGAATCTTGCGACGAAGTGGAGAG
Cyt-C	CAGAGATGTGCCCAGTG CCATAC	GGTGATGCCCTTGGATTTGTTTGC
Actin	GGCTCCTTCCACCATCAA GATCAAG	TTCCAGGACCGGACTCTTCATACTC

### Metabolomic Analysis

Muscle samples (*n* = 6) from the two treatments were harvested and extracted for metabolomic analysis. The differentially expressed metabolites (DEMs) in the muscle tissues of the two treatments were analyzed using a gas chromatograph system coupled to a Pegasus HT time-of-flight mass spectrometer (GC-TOF-MS).

The resulting three-dimensional data comprising the peak number, sample name, and normalized peak area were inputted into the SIMCA 14.1 software package (V14.1, MKS Data Analytics Solutions, Umea, Sweden) and was used to perform principal component analysis (PCA) and orthogonal projections for latent structure-discriminant analysis (OPLS-DA). PCA revealed the distribution of original data. A higher level of group separation was obtained by supervised OPLS-DA to improve the understanding of the variables responsible for classification. For further model validation, sevenfold cross-validation was used to estimate the model’s robustness and predictive ability. Next, Student’s *t*-test (*P* < 0.05) combined with the first principal component of variable importance in projection (VIP) values (VIP > 1) was used to determine the species distribution models (SDMs) among the pairwise comparison groups. The Kyoto Encyclopedia of Genes and Genomes (KEGG)^1^ was used to search for metabolic pathways. A free web-based tool, MetaboAnalyst,^2^ was used to conduct pathway analysis, which uses high-quality KEGG metabolic pathways as the backend knowledge base.

## Results

### Growth Performances, Malondialdehyde and Antioxidant Parameters

**FIGURE 1:**
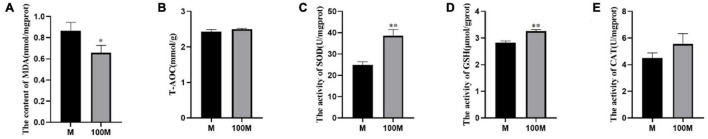
Malondialdehyde (MDA) and antioxidant enzyme activity. **(A)** MDA **(B)** T-AOC **(C)** SOD **(D)** GSH, and **(E)** CAT. Data are expressed as means ± SD (*n* = 3). *<0.05, ^**^<0.01 compared to the control group.

To explore the influence of Axn on growth performances and the antioxidant capacity of *E. carinicauda*, the kits of MDA, T-AOC, GSH, CAT, and SOD were used to prove it. In the muscle, the shrimp in the control group had significantly higher MDA content than those fed with Axn (*P* < 0.05). The activities of T-AOC, GSH, CAT, and SOD in the control group were significantly lower than those in the Axn group (*P* < 0.05) (Figure 1).

Following a 56-day feeding trial period, the FBW of shrimp fed an Axn diet was significantly elevated as compared to shrimp fed a diet (*P* < 0.05; Table 3). Consistently, the control group exhibited lower weight gain (WG) and specific growth rate (SGR) values as compared to those of Axn-fed *E. carinicauda*.

**TABLE 3:** *Exopalaemon carinicauda* growth performance following experimental diet feeding for 56 days.

	FBW (g)	WG (%)	SGR (%)
Control group	0.83 ± 0.08	730 ± 70	3.78 ± 0.01
Astaxanthin group	1.11 ± 0.16*	1010 ± 150*	4.29 ± 0.08*

Data are expressed as means ± SD (n = 3). *<0.05 compared to the control group.

### Transcriptomic Alteration of *Exopalaemon carinicauda* Muscle Affected by Astaxanthin Feeding

**FIGURE 2:**
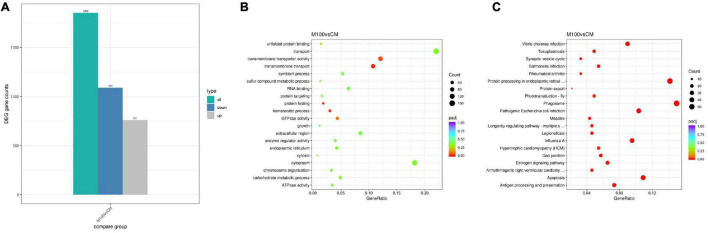
**(A)** Differential expression of *Exopalaemon carinicauda* following Axn feeding. Significantly upregulated and downregulated genes are, respectively, shown in blue and gray. **(B)** The most enriched Gene Ontology (GO) term analysis and function classifications of the transcriptomic responses for *Exopalaemon carinicauda* after feeding Axn. **(C)** Kyoto Encyclopedia of Genes and Genomes pathway analysis of the transcriptomic responses for *E. carinicauda* after feeding Axn.

A total of 1,852 differentially expressed genes (DEGs) were identified in the muscle of the Axn group compared with the control group, with 1,091 genes showing upregulated expression and 761 genes showing downregulated expression (Figure 2A). Raw data were deposited in the Short Read Archive (SRA) of the NCBI with accession numbers of SRX14060554, SRX14060555, SRX14060556, SRX14060557, SRX14060558, and SRX14060559.

Gene Ontology (GO) analysis was used to annotate these DEGs with terms under biological process, cellular component, and molecular function categories for understanding the biological significance of the DEGs. Most of the DEGs were assigned to cell part and membrane part for the cellular component category, the DEGs were mostly associated with cellular and metabolic processes for the biological process category, and most of the DEGs were categorized into binding and catalytic activities for the molecular function category (Figure 2B).

To identify the biochemical pathways influenced by Axn feeding, the KEGG database was used to perform pathway enrichment analysis on the identified DEGs. Of the pathways identified, the most commonly represented class was related to stress and included several subclasses: “Phagosome,” “Protein processing in endoplasmic reticulum,” “Pathogenic *Escherichia coli* infection,” “Antigen processing and presentation,” “Apoptosis,” “Estrogen signaling pathway,” “Gap junction” and “Protein export” (Figure 2C).

### Metabolomics Alteration of *Exopalaemon carinicauda* Muscle Affected by Astaxanthin Feeding

**FIGURE 3:**
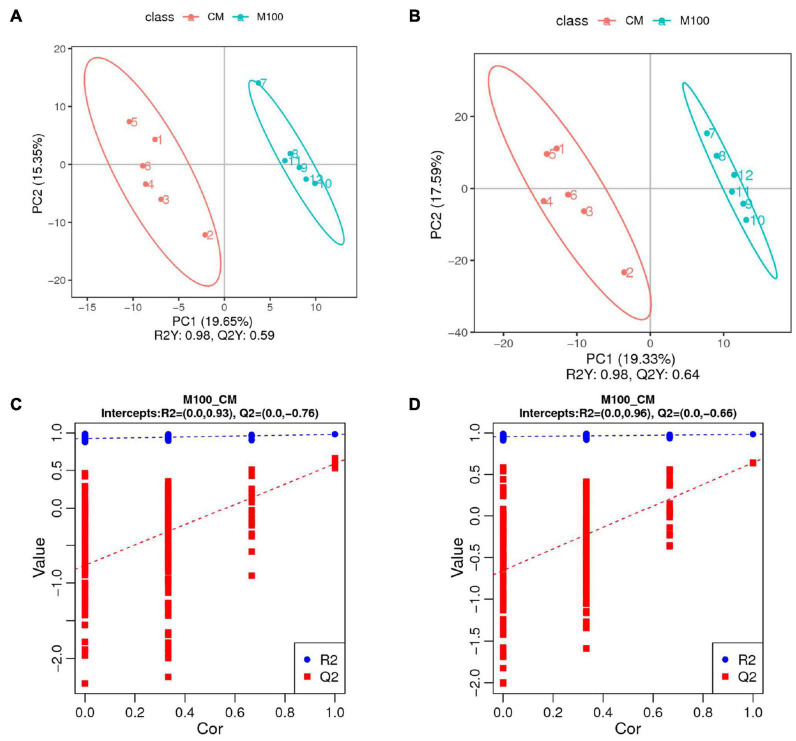
Quality analysis of metabolomic data. **(A)** OPLS-DA (orthogonal partial least-squares-discriminant analysis) score diagram for the positive ion mode. **(B)** OPLS-DA score diagram for the negative ion mode. **(C)** OPLS-DA permutation test for the positive ion mode. **(D)** OPLS-DA permutation test for the negative ion mode.

To investigate the metabolic changes in *E. carinicauda* in response to Axn feeding, an untargeted metabolomic analysis of muscle samples was performed using the UHPLC-Q-TOF-MS platform. A total of 354 negative and 750 positive ion peaks were extracted from the analysis. A total of 136 DEMs were identified in both metabolites, including 62 downregulated metabolites and 74 upregulated metabolites (Table 4). The established OPLS-DA model (model evaluation parameters: positive ion mode: R^2^Y = 0.98 cum, Q^2^Y = 0.64 cum; negative ion mode: R^2^Y = 0.98 cum, Q^2^Y = 0.59 cum) indicated that the model was stable and reliable (Figures 3A,B). Next, a permutation test was used to establish 200 OPLS-DA models in which the order of the categorical variables Y was changed randomly to obtain the R^2^ and Q^2^ values of the stochastic model (Figures 3C,D). From left to right, all Q^2^ points were lower than the original red Q^2^ points on the right, which indicated a robust and reliable model without overfitting. Thus, it is reliable and stable for the test data and instrument analysis system for the experiment.

**TABLE 4:** Differentially expressed metabolites in the shrimp muscle tissue in response to Axn feeding.

Name	Up or down
Alanine	Up
6-Phosphogluconic acid	Up
Glutathione disulfide	Up
Phosphocholine	Up
Glutamine	Up
Leucine	Up
Isoleucine	Up
Vitamin A	Up
Lysops	Up
D-Glucose 6-phosphate	Up
Tyrosine	Up
2-Arachidonoyl glycerol	Up
Lysine	Up
Lysophosphatidic acid	Up
Glutamate	Up
Valine	Up
Ornithine	Up
Threonine	Up
Srine	Up
Phenylalanine	Up
Uric acid	Down
PE	Down
PC	Down
LysoPC	Down
Inosine	Down
Citraconic acid	Down
Lysopc	Down
Arachidic acid	Down

**FIGURE 4:**
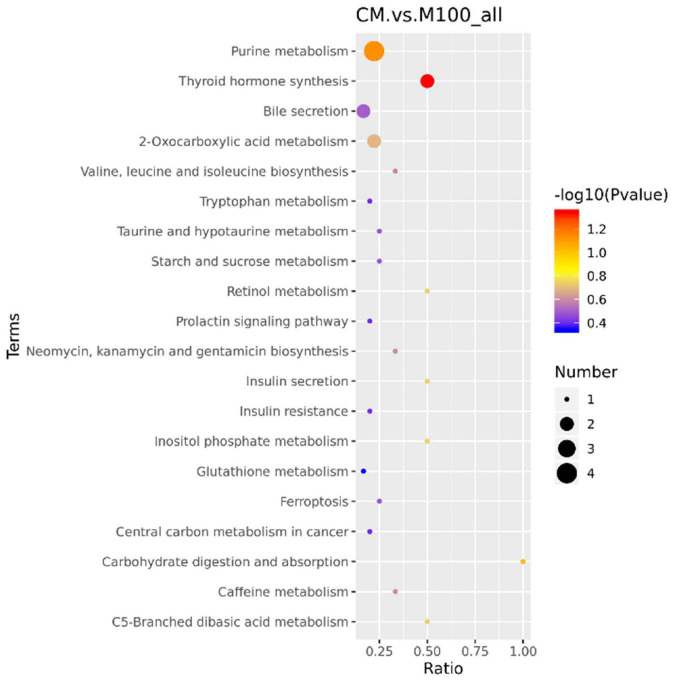
Kyoto Encyclopedia of Genes and Genomes pathways analysis of differentially expressed metabolites in response to Axn. The ordinate represents the top 20 KEGG terms significantly enriched by the DEMs, the abscissa indicates the rich factor between two sampling datasets.

To explore the metabolic pathways that might be affected by Axn feeding, KEGG pathway analysis was used to assign these DEMs to metabolic pathways. The pathway analysis results provided details of the changes in metabolic pathways related to Axn feeding. The most relevant pathways were identified based on a *p*-value < 0.05 and were “Carbohydrate digestion and absorption,” “Inositol phosphate metabolism,” “Valine, leucine and isoleucine biosynthesis,” “Tryptophan metabolism,” “Glutathione metabolism,” “Arginine biosynthesis,” “Biosynthesis of unsaturated fatty acids” (Figure 4).

### Identification of Key Genes and Metabolites Using Multi-Omics Analysis

**FIGURE 5:**
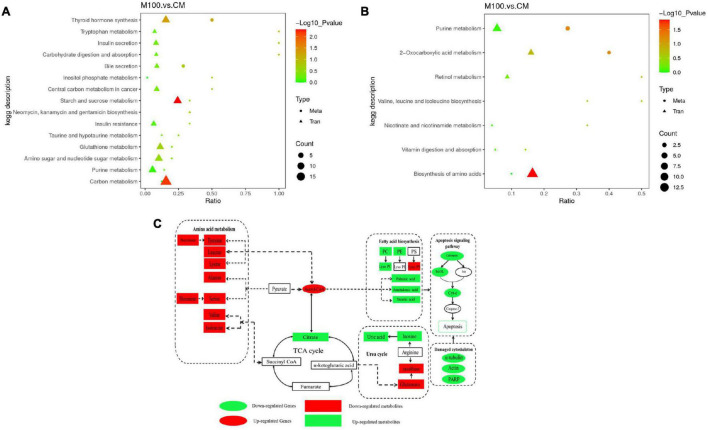
**(A)** Kyoto Encyclopedia of Genes and Genomes pathways analysis of association analysis between transcriptome and metabolome in the positive ion mode. **(B)** Kyoto Encyclopedia of Genes and Genomes pathways analysis of association analysis between transcriptome and metabolome in the negative ion mode. **(C)** Correlation map of genes and metabolites regulated by Axn feeding in the muscle of *Exopalaemon carinicauda*. The altered genes and metabolites are shown by marking the names in red (upregulated) or green (downregulated).

Kyoto Encyclopedia of Genes and Genomes pathway analysis of genes and metabolomics was performed to determine correlations between the transcriptomic and metabolomic data (Figure 5). The analysis showed that the urea cycle, TCA cycle, amino acid metabolism, fatty acid metabolism, and apoptosis signaling pathways were affected by Axn feeding. These pathways are important components of metabolic pathways. Accordingly, the results showed that downregulation of citrate indicates vigorous metabolism of the TCA cycle. Similarly, it was observed that upregulation of most of the DEGs and DEMs was related to amino acid biosynthesis and fatty acid metabolism. Interestingly, the levels of fatty acids, such as arachidonic acid and palmitic acid, were downregulated while inosine was upregulated. These results indicate the importance of these metabolites in energy replenishment.

### Verification of Transcriptomics Data by Quantitative Real-Time Reverse Transcription Polymerase Chain Reaction

**FIGURE 6:**
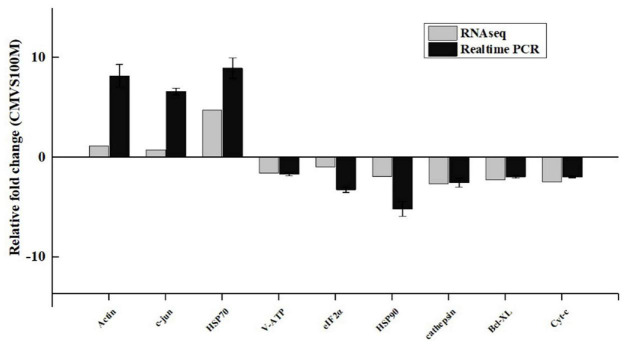
Comparison of gene expression data between RNA sequencing (RNA-Seq) and quantitative real-time reverse transcription PCR (qRT-PCR). Data are presented as mean ± standard deviation (SD) of three replicates.

To further verify the results of the transcriptome-based quantitative analysis, qRT-PCR was performed. The mRNA transcription levels of nine genes, including six downregulated (cathepsin, eIF2α, Cyt-C, V-ATP, HSP90, and Bcl-XL) and three upregulated (Hsp70, c-jun, and Actin), were measured. The expression levels of the genes showed similar trends with the RNA-sequencing (RNA-seq) results, which indicates the reliability and accuracy of the RNA-seq analysis (Figure 6).

## Discussion

Astaxanthin, a new feed additive, has been investigated for its effect on the overall biological processes in various species (Xie et al., 2018, 2020; Yu et al., 2020). Although transcriptome analysis has provided insights into the mechanism of Axn enrichment in *E. carinicauda* (Jin et al., 2021), the research only elucidates the mechanism in the mutant species. The molecular mechanism of Axn feeding in common *E. carinicauda* remains unclear.

In the current study, we analyzed antioxidant enzymes and changes in genes and metabolites involved in Axn feeding. Antioxidant enzyme analysis demonstrated that Axn can improve the antioxidant capacity of *E. carinicauda*. Furthermore, it was confirmed that Axn feeding affected amino acid, fatty acid, and energy metabolism, which may be related to the cytoskeleton and apoptosis.

### Alteration of Amino Acid Metabolism Associated With Astaxanthin Feeding

The results showed that Axn feeding triggered a response involving amino acid metabolism. According to the metabolome data, the levels of most amino acids (e.g., alanine, arginine, glutamic acid, leucine, isoleucine, lysine, aspartic acid, valine, serine, threonine, and phenylalanine) were significantly upregulated in the shrimp after Axn feeding. In addition, the RNA-seq data indicated that Axn feeding induced significant changes in the expression levels of amino acid metabolism-associated genes. Citrate, which is the first intermediate of the TCA cycle, and other TCA cycle intermediates, such as arginine and glutamic acid, are important precursors of α-ketoglutarate, acetyl-CoA, and succinyl-CoA (Wu et al., 2018). Other metabolites, such as isoleucine and leucine, participate in immunity, neurotransmission, protein synthesis, and energy production (Zhang et al., 2017). These results suggest that Axn may induce changes in amino acid metabolism.

Glutamine is an important detoxification substance. The significant increase in glutamine content indicates higher antioxidant activity and higher tolerance when the body is injured. L-glutamate, an abundant free amino acid in the body, is derived from the local synthesis of L-glutamine and Krebs cycle intermediates. Glutamate not only enhances the perception of sweetness and saltiness, but also supports muscle protein synthesis and enhances alterations in immunological responses (Tapiero et al., 2002). In this study, the amount of various amino acids in the muscles of *E. carinicauda* after Axn feeding increased significantly and included not only the amino acids that form the cytoskeleton but also those that enhance flavor. It has been shown that Axn not only improves the anti-apoptotic ability of muscle, but also influences the flavor of *E. carinicauda*.

Glutamate synthesizes glutamine for detoxification and the cytoskeleton and is also used as a raw material for the synthesis of GSH. The results of this study showed that GSH was upregulated. GSH plays a role in signal transduction, gene expression, and apoptosis. Most of the GSH involved in antioxidant defense in cells is utilized by three members of the GSH peroxidase (GPx) family and one peroxiredoxin (Prdx 6). These enzymes catalyze the reduction of H_2_O_2_ by GSH into H_2_O and GSSG (Forman et al., 2009). Based on the results, it is speculated that the content of glutamate may be increased after Axn feeding, which may also lead to an increase in glutathione content. This also means that the antioxidant capacity of *E. carinicauda* was greatly improved as a result of Axn feeding.

### Astaxanthin Feeding-Induced Alterations to Lipid Metabolism

In terms of lipid metabolism, some DEGs and DEMs were downregulated in response to Axn feeding and were associated with unsaturated fatty acid (UFA) biosynthesis. UFAs are indispensable constituents of cellular membranes and are involved in energy metabolism (Hulbert, 2003). Metabolomic analysis of the muscle indicated that the fatty acid biosynthetic and arachidonic acid metabolic pathways were enriched in the Axn group. Levels of palmitic acid, myristic acid, and arachidonic acid (ARA) were decreased in muscles in response to Axn feeding. Furthermore, the levels of certain fatty acids and energy metabolism-related molecules, such as leukotrienes and glucose-6-phosphate (Tallima and El Ridi, 2018), were also increased in Axn-fed shrimp compared with the control group. The levels of UFAs have been reported to increase under adverse environmental stress in white shrimp and Chinese fleshy shrimp (Fan et al., 2019; Meng et al., 2019). The results of the study indicated that the anti-stress ability of the cell membrane of muscle was improved after feeding with Axn and that it was not necessary for many UFAs to participate in cell membrane synthesis.

The cell membrane has a certain fluidity. The levels of long-chain polyUFAs are generally increased to maintain membrane fluidity, in what is termed homeoviscous adaptation. Phosphoglycerides, including lecithin (PC), cephalin (PE), and phosphatidylserine (PS), are important components of the cell membrane and form the basis of cell metabolism, energy metabolism, and signal transmission. Lysophospholipids, the raw material of biomembranes, also participate in the biological processes of most cells (Schulte, 2015; Wang et al., 2020). After Axn feeding, the levels of almost all PEs, PCs, PSs, and LysoPCs decreased. Therefore, it can be inferred that *E. carinicauda* activates metabolic pathways, such as ARA metabolism, to protect the muscle from injury. In addition, the DEMs detected in lipid metabolism was less than that detected in amino acid metabolism. This may be because the protein content of the muscle of *E. carinicauda* is richer than the lipid content of the muscle of *E. carinicauda*.

Interestingly, it was found that the vitamin A content increased after Axn feeding. It is speculated that Axn is converted into vitamin A and stored in white shrimp. When *E. carinicauda* was stimulated by an adverse environment, vitamin A had corresponding physiological effects.

### Cytoskeleton Changes and Apoptosis Induced by Astaxanthin Feeding

Aquatic animals are susceptible to oxidative stress caused by biotic and abiotic factors (Martinez-Alvarez et al., 2005; Lushchak, 2011), which results in the accumulation of reactive oxygen species (ROS) and changes in antioxidant enzymes that lead to tissue damage or cell apoptosis (Lushchak, 2011; Lin et al., 2017). Axn is essential for maintaining proper antioxidant capacity and the health of aquatic organisms to alleviate the negative effects triggered by oxidative stress (Lim et al., 2018). In this study, the activity of antioxidant enzymes was significantly upregulated, which also demonstrates the protective effect of Axn against *E. carinicauda*.

Among the DEGs, it was shown that ACT1, which participates in cytoskeleton remodeling, was upregulated by Axn feeding. Protection against stress was found to be associated with changes in the expression levels of genes related to cytoskeleton remodeling (Xiao et al., 2019). When the body is adversely stimulated, cytoskeleton-related genes are significantly downregulated. When the expression of these genes increase significantly, the anti-stress ability of the body is significantly improved (Long et al., 2013). Thus, changes in ACT1 expression might be involved in Axn feeding; however, the detailed mechanism of the relationship between remodeling of cytoskeleton structure and Axn should be explored in further studies.

In the present study, the expression of cathepsin was significantly decreased after Axn feeding, indicating its potential role in apoptosis. Moreover, cathepsin, which induces upregulation of proapoptotic genes and downregulation of antiapoptotic genes (Li et al., 2016), may cause Cyt-c to be released into the cytoplasm from mitochondria, leading to apoptosis. Similar results were found in a previous study, in which cathepsin and Cyt-c expression decreased significantly after Axn feeding compared with the control, suggesting that Axn participates in the mitochondrial apoptotic pathway (Ren et al., 2020). The antiapoptotic BCL-XL can inhibit the release of Cyt-c from the mitochondria. Thus, it is interesting to note that BCL-XL decreased after Axn feeding. The reason for this may be that Axn improved the anti-stress ability of the shrimp, which lead to the antiapoptotic gene Bcl-XL not being highly expressed in response to adverse environmental stimuli.

## Conclusion

In conclusion, we herein found that the growth performance and antioxidant enzyme activity of *E. carinicauda* fed a diet containing Axn was superior to that of shrimp fed a control diet. There is a significant difference in the transcriptome and metabolome of the *E. carinicauda* muscle after Axn feeding. Transcriptomic and metabolomic analysis revealed 1852 DEGs and 136 DEMs. Interaction analysis indicated that the DEGs and DEMs were mostly involved in the metabolism of amino acids and UFAs. The results of this study provide novel insights into the response of the muscle of *E. carinicauda* after Axn feeding.

## Data Availability Statement

The data presented in the study are deposited in the National Center for Biotechnology Information (NCBI) repository, accession number PRJNA803954.

## Ethics Statement

The animal study was reviewed and approved by The Experimental Animal Ethics Committee, Yellow Sea Fisheries Research Institute, Chinese Academy of Fishery Sciences, China approved the present study.

## Author Contributions

JaL and WL: conceptualization. JW, JaL, and PL: methodology. WL: software, validation, investigation, data curation, writing original draft preparation, and visualization. JW and WL: formal analysis. JtL: resources, project administration, and funding acquisition. JW, JaL, and FZ: writing review and editing. JaL and PL: supervision. All authors have read and agreed to the published version of the manuscript.

## Conflict of Interest

The authors declare that the research was conducted in the absence of any commercial or financial relationships that could be construed as a potential conflict of interest.

## Publisher’s Note

All claims expressed in this article are solely those of the authors and do not necessarily represent those of their affiliated organizations, or those of the publisher, the editors and the reviewers. Any product that may be evaluated in this article, or claim that may be made by its manufacturer, is not guaranteed or endorsed by the publisher.

## Footnotes

1
http://www.genome.jp/kegg/

2
http://www.metaboanalyst.ca

## References

[B1] Albrektsen S. Ostbye T. K. Pedersen M. Ytteborg E. Ruyter B. Ytrestoyl T. (2018). Dietary impacts of sulphuric acid extracted fish bone compounds on astaxanthin utilization and muscle quality in Atlantic salmon (*Salmo salar*). *Aquaculture* 495 255–266.

[B2] Fakhri S. Abbaszadeh F. Dargahi L. Jorjani M. (2018). Astaxanthin: a mechanistic review on its biological activities and health benefits. *Pharmacol. Res.* 136 1–20. 10.1016/j.phrs.2018.08.012 30121358

[B3] Fan L. F. Wang L. Wang Z. L. (2019). Proteomic characterization of the hepatopancreas in the Pacific white shrimp *Litopenaeus vannamei* under cold stress: revealing the organism homeostasis mechanism. *Fish Shellfish Immunol.* 92 438–449. 10.1016/j.fsi.2019.06.037 31229644

[B4] Fang N. Wang C. K. Liu X. F. Zhao X. Liu Y. H. Liu X. M. (2019). De novo synthesis of astaxanthin: from organisms to genes. *Trends Food Sci. Technol.* 92 162–171. 10.1111/mec.12781 24803335

[B5] Forman H. J. Zhang H. Q. Rinna A. (2009). Glutathione: overview of its protective roles, measurement, and biosynthesis. *Mol. Aspects Med.* 30 1–12. 10.1016/j.mam.2008.08.006 18796312 PMC2696075

[B6] Ge Q. Q. Li J. Wang J. J. Li J. T. Ge H. X. Zhai Q. Q. (2017). Transcriptome analysis of the hepatopancreas in *Exopalaemon carinicauda* infected with an AHPND-causing strain of *Vibrio parahaemolyticus*. *Fish Shellfish Immunol.* 67 620–633. 10.1016/j.fsi.2017.06.047 28648882

[B7] Hulbert A. J. (2003). Life, death and membrane bilayers. *J. Exp. Biol.* 206 2303–2311. 10.1242/jeb.00399 12796449

[B8] Huo D. Sun L. N. Zhang L. B. Ru X. S. Liu S. L. Yang H. S. (2019). Metabolome responses of the sea cucumber *Apostichopus japonicus* to multiple environmental stresses: heat and hypoxia. *Mar. Pollut. Bull.* 138 407–420. 10.1016/j.marpolbul.2018.11.063 30660290

[B9] Jin Y. Li S. A. Yu Y. Zhang C. S. Zhang X. J. Li F. H. (2021). Transcriptome analysis provides insights into the mechanism of astaxanthin enrichment in a mutant of the ridgetail white prawn *Exopalaemon carinicauda*. *Genes* 12:618. 10.3390/genes12050618 33919403 PMC8143343

[B10] Li M. Y. Sun L. Niu X. T. Chen X. M. Tian J. X. Kong Y. D. (2019). Astaxanthin protects lipopolysaccharide-induced inflammatory response in *Channa argus* through inhibiting NF-kappa B and MAPKs signaling pathways. *Fish Shellfish Immunol.* 86 280–286. 10.1016/j.fsi.2018.11.011 30448447

[B11] Li Y. H. Wei L. Cao J. R. Qiu L. G. Jiang X. Li P. (2016). Oxidative stress, DNA damage and antioxidant enzyme activities in the pacific white shrimp (*Litopenaeus vannarnei*) when exposed to hypoxia and reoxygenation. *Chemosphere* 144 234–240. 10.1016/j.chemosphere.2015.08.051 26363325

[B12] Lim K. C. Yusoff F. M. Shariff M. Kamarudin M. S. (2018). Astaxanthin as feed supplement in aquatic animals. *Rev. Aquac.* 10 738–773.

[B13] Lin Y. Huang J. J. Dahms H. U. Zhen J. J. Ying X. P. (2017). Cell damage and apoptosis in the hepatopancreas of *Eriocheir sinensis* induced by cadmium. *Aquat. Toxicol.* 190 190–198. 10.1016/j.aquatox.2017.07.008 28750221

[B14] Long Y. Song G. L. Yan J. J. He X. Z. Li Q. Cui Z. B. (2013). Transcriptomic characterization of cold acclimation in larval zebrafish. *BMC Genomics* 14:612. 10.1186/1471-2164-14-612 24024969 PMC3847098

[B15] Lushchak V. I. (2011). Environmentally induced oxidative stress in aquatic animals. *Aquat. Toxicol.* 101 13–30. 10.1016/j.aquatox.2010.10.006 21074869

[B16] Martinez-Alvarez R. M. Morales A. E. Sanz A. (2005). Antioxidant defenses in fish: biotic and abiotic factors. *Rev. Fish Biol. Fish.* 15 75–88. 10.1007/s11160-005-7846-4

[B17] Mattei R. Polotow T. G. Vardaris C. V. Guerra B. A. Leite J. R. Otton R. (2011). Astaxanthin limits fish oil-related oxidative insult in the anterior forebrain of Wistar rats: putative anxiolytic effects? *Pharmacol. Biochem. Behav.* 99 349–355. 10.1016/j.pbb.2011.05.009 21619892

[B18] May M. (2017). Big data, big picture: metabolomics meets systems biology. *Science* 356 646–648. 10.1126/science.356.6338.646

[B19] Meng X. H. Dong L. J. Shi X. L. Li X. P. Sui J. Luo K. (2019). Screening of the candidate genes related to low-temperature tolerance of *Fenneropenaeus chinensis* based on high-throughput transcriptome sequencing. *PLoS One* 14:e0211182. 10.1371/journal.pone.0211182 30958828 PMC6453463

[B20] Ning M. X. Wei P. P. Shen H. Wan X. H. Jin M. J. Li X. Q. (2019). Proteomic and metabolomic responses in hepatopancreas of whiteleg shrimp *Litopenaeus vannamei* infected by microsporidian *Enterocytozoon hepatopenaei*. *Fish Shellfish Immunol.* 87 534–545. 10.1016/j.fsi.2019.01.051 30721776

[B21] Ren X. Y. Yu Z. X. Xu Y. Zhang Y. B. Mu C. M. Liu P. (2020). Integrated transcriptomic and metabolomic responses in the hepatopancreas of kuruma shrimp (*Marsupenaeus japonicus*) under cold stress. *Ecotoxicol. Environ. Saf.* 206:111360. 10.1016/j.ecoenv.2020.111360 32979723

[B22] Schulte P. M. (2015). The effects of temperature on aerobic metabolism: towards a mechanistic understanding of the responses of ectotherms to a changing environment. *J. Exp. Biol.* 218 1856–1866. 10.1242/jeb.118851 26085663

[B23] Sun J. Zhou Q. X. Hu X. G. (2019). Integrating multi-omics and regular analyses identifies the molecular responses of zebrafish brains to graphene oxide: perspectives in environmental criteria. *Ecotoxicol. Environ. Saf.* 180 269–279. 10.1016/j.ecoenv.2019.05.011 31100591

[B24] Tallima H. El Ridi R. (2018). Arachidonic acid: physiological roles and potential health benefits – a review. *J. Adv. Res.* 11 33–41. 10.1016/j.jare.2017.11.004 30034874 PMC6052655

[B25] Tapiero H. Mathe G. Couvreur P. Tew K. D. (2002). Dossier: free amino acids in human health and pathologies – II. Glutamine and glutamate. *Biomed. Pharmacother.* 56 446–457. 12481981 10.1016/s0753-3322(02)00285-8

[B26] Wang L. Huang X. L. Sun W. L. Too H. Z. Laserna A. K. C. Li S. F. Y. (2020). A global metabolomic insight into the oxidative stress and membrane damage of copper oxide nanoparticles and microparticles on microalga *Chlorella vulgaris*. *Environ. Pollut.* 258:113647. 10.1016/j.envpol.2019.113647 31810715

[B27] Wen X. Hu Y. D. Zhang X. Y. Wei X. Z. Wang T. Yin S. W. (2019). Integrated application of multi-omics provides insights into cold stress responses in pufferfish *Takifugu fasciatus*. *BMC Genomics* 20:563. 10.1186/s12864-019-5915-7 31286856 PMC6615287

[B28] Wu Z. Jin L. Zheng W. Zhang C. Zhang L. Chen Y. (2018). NMR-based serum metabolomics study reveals a innovative diagnostic model for missed abortion. *Biochem. Biophys. Res. Commun.* 496 679–685. 10.1016/j.bbrc.2018.01.096 29353036

[B29] Xiao J. Li Q. Y. Tu J. P. Chen X. L. Chen X. H. Liu Q. Y. (2019). Stress response and tolerance mechanisms of ammonia exposure based on transcriptomics and metabolomics in *Litopenaeus vannamei*. *Ecotoxicol. Environ. Saf.* 180 491–500. 10.1016/j.ecoenv.2019.05.029 31121556

[B30] Xie S. W. Fang W. P. Wei D. Liu Y. J. Yin P. Niu J. (2018). Dietary supplementation of *Haematococcus pluvialis* improved the immune capacity and low salinity tolerance ability of post-larval white shrimp, *Litopenaeus vannamei*. *Fish Shellfish Immunol.* 80 452–457. 10.1016/j.fsi.2018.06.039 29933110

[B31] Xie S. W. Yin P. Tian L. X. Yu Y. Y. Liu Y. J. Niu J. (2020). Dietary supplementation of astaxanthin improved the growth performance, antioxidant ability and immune response of juvenile largemouth bass (*Micropterus salmoides*) fed high-fat diet. *Mar. Drugs* 18:642. 10.3390/md18120642 33333811 PMC7765211

[B32] Xu W. J. Xie J. J. Shi H. Li C. W. (2010). Hematodinium infections in cultured ridgetail white prawns, *Exopalaemon carinicauda*, in eastern China. *Aquaculture* 300 25–31. 10.1016/j.aquaculture.2009.12.024

[B33] Yu Y. Y. Liu Y. Yin P. Zhou W. W. Tian L. X. Liu Y. J. (2020). Astaxanthin attenuates fish oil-related hepatotoxicity and oxidative insult in juvenile Pacific white shrimp (*Litopenaeus vannamei*). *Mar. Drugs* 18:218. 10.3390/md18040218 32316590 PMC7230248

[B34] Zhang C. S. Su F. Li S. H. Yu Y. Xiang J. H. Liu J. G. (2018). Isolation and identification of the main carotenoid pigment from a new variety of the ridgetail white prawn *Exopalaemon carinicauda*. *Food Chem.* 269 450–454. 10.1016/j.foodchem.2018.06.143 30100459

[B35] Zhang S. Zeng X. Ren M. Mao X. Qiao S. (2017). Novel metabolic and physiological functions of branched chain amino acids: a review. *J. Anim. Sci. Biotechnol.* 8:10. 10.1186/s40104-016-0139-z 28127425 PMC5260006

